# The role of self-endangering cognitions between long-term care nurses' altruistic job motives and exhaustion

**DOI:** 10.3389/frhs.2023.1100225

**Published:** 2023-08-23

**Authors:** Lara L. Eder, Bertolt Meyer

**Affiliations:** Department of Psychology, Chemnitz University of Technology, Chemnitz, Germany

**Keywords:** self-esteem, self-endangering behavior, altruim, coping, burnout, self-endangering cognitions, nursing

## Abstract

**Background:**

Due to demographic change and staff shortages nurses suffer under high work strain. As a consequence, caregivers' absenteeism due to mental stress, in particular burnout, is high. To explain the development of nurses' burnout more research is needed on nurses' individual resources and coping strategies. Self-endangering is a potentially harmful coping strategy.

**Objective:**

To expand the perspective of the Job Demand-Resources Model by including caregivers' intraindividual resources and the coping construct of self-endangering as a mediator between personal resources and nurses' emotional exhaustion.

**Methods:**

A longitudinal questionnaire survey was conducted between July 2020—March 2021 among nurses in long-term care in Germany. The final analysis sample consisted of *wave 1* = 416 and *wave 1,2_ _*= 50. Data were analysed by a multiverse analytic strategy using regression analysis with measurement repetition and cross-lagged-panel design for waves one and two. Variables used for regression analysis and cross-lagged-panel were: Independent variables: An altruistic job motivation, team identification and self-esteem, dependent variables: Exhaustion and disengagement, and mediators: Self-endangering cognitions and behavior tendencies.

**Results:**

A highly altruistic job motivation leads to more self-endangering cognitions and to more self-endangering behavior tendencies. Mixed model analysis and cross-sectional path analysis confirmed mediation effects from altruism over self-endangering to exhaustion.

**Conclusion:**

Our findings are at odds with some research findings about altruism in nursing, such that too much altruism can lead to harmful self-endangering. We also introduce a new instrument to capture self-endangering in nursing care. Future research should investigate various facets of self-endangering in nursing. We assume that leadership behavior could have influence on self-endangering. New health policy structures are needed to improve working conditions in nursing and thus prevent self-endangering.

## Introduction

Even before the coronavirus disease (COVID-19) pandemic, there was a known global shortage of nursing staff in the health care system (1). Nevertheless, the pandemic has made it even clearer: The staff shortage in nursing as a result of overwork and poor working conditions is enormous. Further, the pandemic put nurses under overwhelming pressure and in consequence has led to increasing numbers of nurses leaving the profession (2). As a result of nurses' working conditions, the increased workload, and extra work due to a lack of staff, burnout is rampant (3), and there is mass trauma among nurses (4). In addition, the number of people in need of care has been rising for years; this development alone has resulted in an increasing demand for nurses in the coming decades (1).

Due to these and future developments in the health care system, the importance of a motivated and healthy nursing staff is greater than ever, because this is the only way to meet the demographic challenges. What can be done to protect the mental health of remaining nurses and to maintain their ability to work?

Shift work, frequent overtime and filling in on days off, and the difficulty of separating oneself from work (5) are just a few examples of the highly demanding working conditions in nursing. Due to this, nurses need to have very high levels of self-directed action, communication skills, and self-organization abilities (6), which is why both individual prevention and organization of work are particularly important in protecting nurses' health. Various research projects have already developed prevention action guidelines and training programs for individual resources (7) and organization structures (8) in everyday nursing care, such as in Germany “Working happily and healthy in geriatric care” (8), “Healthy working conditions in care facilities” (8), “Care for caregivers: Development and anchoring of an empathy-based relief concept in care work (empCARE)” (7). However, considering the still high number of stresses, absenteeism from work due to mental disorders (9), and career interruptions (2), the question arises as to what care institutions are aware of, using, and successfully implementing these programs and guidelines. We therefore argue that the continuous high level of nurses' burnout (3) as a result of high demands calls for new strategies in dealing with the challenges for nurses and care organizations that go beyond previous findings in the area of demands and resources.

Regarding individual resources, studies have found that organizational interventions that ignore nurses' individual factors cannot sustainably reduce exhaustion and that coping strategies and improving nurses' resilience are important for decreasing burnout (10). As for other individual factors, recent studies found that although being altruistically motivated (11) or highly identified with the team (12) leads to improvements in nurses' burnout, qualitative results show divergent trends (13).

By considering self-endangering as an important coping strategy in nursing, we see great potential for a broader perspective for nurses' training programs (13). Self-endangering is a coping strategy when employees are confronted with high workloads and demands for self-organization; the strategy is functional for reaching work goals but dysfunctional for health (14). There are only few studies available on the long-term effects of self-endangering, but Baeriswyl et al. (2014) (15) found detrimental effects on teachers' well-being, and Knecht et al. (2017) (16) found that self-endangering work behaviors might partly explain the association between work demands and exhaustion.

We hope to contribute to research on nurses' occupational health by expanding the job demands-resources model (JD-R) (17) to include personal characteristics and the construct of self-endangering as a mediator between personal resources and nurses' emotional exhaustion. We thus hope that our results can also help make interventions for caregivers more effective by addressing individual characteristics and needs and strengthening coping strategies.

We investigated our hypotheses in a three-wave questionnaire survey of nurses in long-term care in Germany from June 2020 to April 2021. For this, we contacted nursing homes in Germany about participating in the study and in addition shared the online questionnaire link in various social media nursing groups.

For predicting employees' exhaustion, the JD-R theory appears relevant. According to the JD-R, job characteristics consist of two dimensions, job resources and job demands (18). Job demands in nursing are in general work pressure, work time or staff capacities, and emotional demands, such as having to deal with death and dying or generally to interact with patients and relatives (19). Meaningful job resources in nursing are social relations, management support, decision latitude, and task significance (19).

As personality patterns and coping strategies (20, 21) have a potentially great influence on the way people deal with stress, such that based on the transactional stress theory, coping reactions mediate the effect of stress on well-being (22), a nursing specific demand-resources model emphasizes the effects of individual characteristics and resources and the coping strategy self-endangering (13).

### Antecedents of self-endangering in nursing care

Self-endangering work behavior can be understood as a coping strategy in highly demanding working situations (14). It is defined as “actions that aim to deal with work-related demands but simultaneously increase the likelihood of health problems and impede necessary recovery from work-related stress” (14).

We understand self-endangering in nursing as self-sacrificing cognitions and behavior, such that nurses have their “own inner beliefs with regard to having a moral obligation to fill in for colleagues at the expense of their own health” and in consequence have a “diminished ability to say no when asked to fill in or to do work overtime” (13). Therefore, self-endangering in nursing is a coping strategy for dealing with high demands in nursing care based on nurses' individual attitudes and values and personality traits, which in the long term worsens psychological well-being.

Although an interest in professional success justifies self-endangering behavior in the previous research (23), in the field of elderly care we posit that job motives and individual attitudes and values are the reasons for nurses' self-endangering. Altruism is nurses' most important work value (24–26), but recent research postulates improvements in nurses' burnout symptoms with altruistic work values (11). However, studies also found that in the sense of moral stress (27), the gap between professional ideals and working reality may lead to burnout (28, 29). We assume this gap between nurses' altruistic motives and the working conditions that do not allow helping others adequately. We conclude that: altruistic job motivation is highly pronounced in nurses (24–26), and the highly demanding working situation leads nurses to neglect their own health and go beyond their own boundaries because they focus on others and helping others and not themselves.

Based on these findings, we formulated the following hypotheses:

**Hypothesis 1a:** Altruistic job motivation affects self-endangering cognitions such that more altruism increases self-endangering cognitions.
**Hypothesis 1b:** Altruistic job motivation affects self-endangering behavior such that more altruism increases self-endangering behavior.

As a result of self-endangering developments in organizations, employees want their colleagues to reach the same degree of performance and engagement when working goals are based on team levels (23). We understand the working goal in nursing to be the patients' healing or caring process, so in consequence, due to shift systems and interdisciplinary cooperation, this can only be done through the joint work of the team. Here, the team or organization is the nurses' social identification (30), which is defined as “the positive emotional valuation of the relationship between self and ingroup” (31). As nurses often have to work 12 days in a row, they spend a lot of time with their teammates, and as the work content is often emotionally demanding, we assume a special connection between colleagues. And as a result of difficult working conditions, failures in care are very common (9). Peer pressure in cases of absences is especially high when organizations give employees the feeling that illnesses are not tolerated or when they do not provide temporary staff to fill in for personnel (23). We postulate that, when a nurse who identifies strongly with the team is asked to fill in, the probability that they cannot say no is very high, because, first, out of the emotional valuation of the relationship they feel a strong responsibility for their colleagues' well-being and, second, they might think that filling in for others is a necessary part of the team relationship. Of course, we could also assume an inverted u-shaped course at this point, such that low and high levels of team identification result in low levels of self-endangering and only a middle level of team identification results in high levels of self-endangering. However, due to social identification theories, we assume a linear progression and formulated the following hypotheses:

**Hypothesis 2a:** Team identification affects self-endangering cognitions such that more team-identification increases self-endangering cognitions.
**Hypothesis 2b:** Team identification affects self-endangering behavior such that more team-identification increases self-endangering behavior.

As described above, we posit that social identification plays a special role in the context of nursing. In addition to nurses' identification with their team, we consider social identity, as group-relevant behaviors are associated with an individual's self-definition (32). According to social identity theory, people strive for the establishment or enhancement of positive self-esteem and a part of a person's self-concept is based upon the person's group memberships (32). Based on these assumptions, we postulate that caregivers strive for positive self-esteem by being part of caring memberships characterized by relationships with residents and colleagues. If self-esteem is generally rather low, we see a potential risk for the nurses' well- being, as it could drive them to find their self-esteem affirmation in this group affiliation. If part of the nurses' work motivation is to strengthen their own self-esteem, it is obvious from our point of view that nurses cannot say no and have a guilty conscience if they do not fill in in cases of absences. A study in Poland found that self-esteem is a predictor for professional burnout in nursing and a key factor for preventing nurses' burnout (33). In line with previous research we therefore argue that the more nurses use their work to enhance their self-worth and feel validated, the more they will be willing to exceed their own health limits and show self-endangering:

**Hypothesis 3a:** Self-esteem affects self-endangering cognitions such that less self-esteem increases self-endangering cognitions.
**Hypothesis 3b:** Self-esteem affects self-endangering behavior such that less self-esteem increases self-endangering behavior.

### Self-endangering and burnout in nursing care

Due to ongoing demographic changes and the additional shortage of skilled workers in nursing, the ever-decreasing number of existing nurses must care for the ever-increasing group of people requiring nursing care (34). In the long term, this intensification of work leads to work strain, so it is not surprising that a study revealed “body postures, handling heavy loads, time pressure, deadlines and pressure to perform as the main burdens” of nurses in geriatric care (35). Nurses’ objective workload and their subjective perception of stress are above average compared to other occupational groups (28). Recent studies revealed that burnout is rampart among nurses (3, 36, 37), especially nurses in long-term care (38).

Based on the Oldenburger Burnout Inventory (OLBI) (39) the construct of burnout includes two core dimensions—exhaustion and disengagement from work—and covers affective, physical, and cognitive aspects. As we define self-endangering cognitions as “the nurses’ own inner beliefs with regard to having a moral obligation to fill in for colleagues at the expense of their own health” (13) and behavior “as the nurses’ missing ability to say no when asked to fill in or to do work overtime” (13), we postulate that nurses tend to go beyond their boundaries when filling in for others or doing extra work and that this is harmful to their health. We assume that this results in the long term in emotional exhaustion, as personal resources are depleted:

**Hypothesis 4a:** Self-endangering cognitions affect emotional exhaustion such that more self-endangering cognitions increase exhaustion.
**Hypothesis 4b:** Self-endangering behavior affects emotional exhaustion such that more self-endangering behavior increases exhaustion.

Work values influence the emotional exhaustion dimension of burnout (40), in that altruistic work values improve nurses' burnout symptoms (11). In contrast, we also found evidence for negative effects of being highly altruistically motivated or strongly identified with the team in the context of nursing (13). Although being altruistically motivated may lead to a high sense of meaning and value at work (41), we see altruistic job motivation more as a risk factor, in the sense that caregivers go beyond their own limits (self-endangering) and become exhausted in the long term.

As nurses frequently practice self-sacrificing (42–44) and this is associated with burnout (43), the question arises to why nurses show self-endangering so frequently when it is so harmful to their health. We see great potential in clarifying these questions and contradictions by evaluating self-endangering as a mediator in the context of nurses’ individual resources and demands and burnout. We formulated the following hypotheses:

**Hypothesis 5a:** Self-endangering cognitions mediate the relationship between altruistic job values and burnout.
**Hypothesis 5b:** Self-endangering behavior mediates the relationship between altruistic job values and burnout.

In line with previous findings on the positive effect of altruistic work values, team identification is also positively associated with health (12). The effect is particularly positive when employees help others cope with stressful events and thereby reduce their negative experience of stress (12), which we assume is especially the case in caring professions. Contrary to these findings, we found indications for a negative impact, such that strong identification with the team leads to self-endangering in highly demanding working situations and this leads in the long term to emotional exhaustion. We therefore hypothesized:

**Hypothesis 6a:** Self-endangering cognitions mediate the relationship between team identification and burnout.
**Hypothesis 6b:** Self-endangering behavior mediates the relationship between team identification and burnout.

Although to our knowledge no study has investigated the concept of self-endangering in nursing, self-sacrificing practices in nursing is probably frequent (42–44) and there is an association between nurses' self-sacrificing and burnout (43). It therefore seems necessary to take a closer look at the interrelationships in quantitative terms.

## Methods

### Sample

We collected data from nurses working in nursing homes and outpatient care services located in Germany in three follow-up waves over a period of nine months from June 2020 to March 2021. For this purpose, we recruited nursing homes for onsite paper-pencil surveys, and we also shared the link to an online survey in social media, e.g., facebook nursing groups and LinkedIn. Each participant created a unique ID and we tracked them over all three measurement points, so participants were the same over all three waves.

The original dataset consisted of 426 individuals at the first wave (starting in June 2020), 52 at the second (starting in November 2020) and 35 at the third wave (starting in January 2021). After we cleaned the data for missing code allocation information and double participation, the analysis sample consisted of *wave 1* = 416, *wave 1,2_ _*= 50, *wave 1,2,3* = 26.

The sample description below refers to the 416 participants who completed the first wave (Table 1). The majority of participants were women (85.33%), and nearly 60% of participants had at least one child. The majority of participants were aged 36–40 years; 87.5% were nurses, and of these 75% were registered nurses. More than half worked in stationary care, and most had worked for 3–5 years at their current organization. The majority of participants had worked for more than 10 years in their profession; 29.80% had a leadership position in the nursing home.

**Table 1:** Sample variables from measurement time 1.

Variable	*N* (*N* total T1 = 416)
Female sex	355
Having a child	250
Age	
15–20 years	10
21–25 years	42
26–30 years	57
31–35 years	58
36–40 years	68
41–45 years	42
46–50 years	37
51–55 years	43
56–60 years	36
61–65 years	14
➢ 65 years	4
Profession*	
Nurse	364
Assistant Social Care	20
Professional Social Care	7
In job training	44
Nursing profession group*	
Registered nurse	273
Nursing assistant (1 year certified)	33
Nursing assistant	32
Care setting*	
Stationary care setting	218
Outpatient care setting	94
Duration of nursing home affiliation*	
0–2 years	108
3–5 years	124
6–10 years	69
➢ 10 years	98
Duration of profession duration*	
0–2 years	22
3–5 years	81
6–10 years	83
➢ 10 years	212
Leadership position	124

* These variables include missing data.

### Procedure

We developed a paper-pencil questionnaire as well as an online version and received ethical approval from University's IRB (institutional review board). We used a voluntary response sampling method, such that we contacted several nursing homes in Germany that we knew from various care projects and asked for their participation. Participating nursing homes could choose whether to use the paper-pencil or the online questionnaire. If the paper-pencil version was requested, we provided a locked box for collecting the questionnaires. The nursing homes' employees could voluntarily decide whether they wanted to participate in the survey.

In addition, we published the online link in multiple social media platforms, e.g., Facebook nursing groups, LinkedIn, and Xing. We informed participants about the aims and background of the study and, in the online version, about the university's privacy and data protection policy. The policy conforms to European and German data protection regulations and was deposited online for participants. Before starting the survey, participants had to give their consent and confirm that they were at least 18 years old. Each participant was asked to generate a pseudonym code. Upon finishing the questionnaire, participants were asked to voluntarily provide their email address for participating in further follow-ups. We stored the email addresses independently from the survey data. All questionnaires were time-stamped.

### Measures

#### Burnout

We assessed burnout with the Oldenburger Burnout Inventory (OLBI) (39) using the exhaustion and disengagement dimensions. The OLBI consists of 16 items; eight items measure the exhaustion dimension of burnout, and eight items measure the disengagement dimension. Responses ranged from 1 (*strong rejection*) to 4 (*strong approval*). Internal validity for exhaustion was high (8 items, *ω_total1,2,3_*_ _= .90, .93, .94). Internal validity for disengagement was high (8 items, *ω_total1,2,3_*_ _= .88, .90, .93). To allow easy interpretation of the results we recoded the inverted items.

#### Self-endangering

Based on our experiences in qualitative research on self-self-endangering in nursing (blinded for peer review) we developed 14 items for self-endangering behavior that we divided into five facets: behavior trends (4 items, e.g., “If a duty shift needs to be covered, I would step in to save my colleagues’ weekend/day off.”), behavior (2 items, e.g., “In the last four weeks I have been asked to fill in for other colleagues”), behavior towards whom (2 items, e.g., “I find it difficult to say no to my superiors when I have to stand in for other colleagues”), distance (2 items, e.g., “I have a hard time saying no on the phone when asked if I can fill in”), and expectations of colleagues (4 items, e.g., “If a service needs to be covered, I expect my colleagues to step in to make sure the residents are well taken care of”). Responses ranged from 1 (*not true at all*) to 5 (*totally true*). Internal validity for self-endangering behavior was high (3items, *ω_total1,2,3_*_ _= .80, .86, .86).

To measure self-endangering cognitions we developed three items for self-endangering cognitions, e.g., “I feel guilty towards my colleagues if I do not fill in”. Internal validity was high (3 items, *ω_total1,2,3_*_ _= .75, .73, .84).

#### Altruistic values

We assessed altruistic job values using the adapted short version of the Dutch version of the Work Importance Study instrument developed by Coetsier and Claes (1990) (45), e.g., “I think it's important to have a job where I can help other people”. Responses ranged from 1 (*totally unimportant*) to 5 (*totally important*). Internal validity was high (7 items, *ω_total1,2,3_*_ _= .88, .90, .91).

#### Identification with the team, the organization, the profession, occupational activity, and the leader

We assessed identification (with the team, organization, profession, occupational activity, and the leader) with the Four Item Measure of Social Identification (FISI) (31), e.g., “I identify with my team.” Responses ranged from 1 (*not at all true*) to 7 (*very true of me*). Internal validity was high (6 items, *ω_total1,2,3_*_ _= .90, .92, .90).

#### Self-worth

We assessed self-worth with the German version of the Single-Item Self-Esteem Scale (G- SISE) (46), e.g., “I have high self-esteem” .Responses ranged from 1 (*not at all true*) to 5 (*very true of me*).

### Exploratory and confirmatory factor analysis

As recommended in Standards for Educational and Psychological Testing (47), we performed exploratory factor analysis (EFA) and confirmatory factor analysis (CFA). We therefore randomly split the dataset from wave one into subsets A (*N* = 200) and B (*N* = 226). We used subset A for conducting the exploratory and confirmatory factor analysis and subset B for testing our hypotheses.

### Exploratory factor analysis

For determining the numbers of factors, we used parallel analysis (PA) and the comparison data approach (CD), as Goretzko et al. (2021) (48) recommended avoiding MAP-test or Kaiser-Guttmann rule as a criterion. The PA using minimum rank factor analysis (MRFA) (49) resulted in three factors. The CD approach (50) suggested six factors. Due to the different results, we calculated analyses for both numbers of factors and subsequently compared the respective model fit.

As the data did not meet the criteria for normally distributed data, we used EFA using weighted least squares (WLS) (48). As the factors were assumed to be correlated, we used oblique rotation methods (51), e.g., oblimin and promax (48). The five factors solution (RMSEA 90% interval = 0.122–0.154) showed a better fit than the six factors solution (RMSEA 90% interval = 0.122–0.160) and the three factors solution (RMSEA 90% interval = 0.167–0.195).

The analysis for the five factors solution yielded a structure with item loadings from 0.52 to 0.96. Each item highly loaded on one factor, whereas one item cross loaded above 0.32 on two factors. We therefore deleted this item for rerunning EFA (52). Further analyses showed additional cross loadings above.32, so we deleted two more items. After deleting the three items, the PA using minimum rank factor analysis (MRFA) (49) resulted in three factors. The comparison data approach (50) suggested four factors. The four factors solution (RMSEA 90% interval = 0.114–0.157) showed a better fit than the three factors solution (RMSEA 90% interval = 0.157–0.193). The analysis for the four factors solution yielded a structure with item loadings from 0.50 to 0.96, and each item highly loaded on one factor (Table 2). Table 2 presents factor intercorrelations.

**Table 2:** Intercorrelations among the factors of self-endangering and exploratory factor analysis using weighted least squares and oblimin rotation: factor loadings of the four factors of self-endangering (after deletion of three items due to crossloadings above 32).

	Factor	F1	F2	F3	F4	*h* ^2^
Intercorrelations						
F2		.31				
F3		.20	.18			
F4		.34	.47	.20		
Factor loadings						
F1: Self-endangering cognitions	1. I find it difficult to say no to my leaders when I have to fill in for colleagues.	**0**.**84**	0.02	0.01	0.08	0.78
	2. I find it difficult to say no to my colleagues when I have to fill in for them.	**0**.**88**	0.03	0.05	−0.08	0.77
	3. I have a hard time saying no on the phone when asked if I can fill in.	**0**.**92**	−0.04	−0.05	0.07	0.85
	4. I find it difficult in personal contact (e.g., directly at work) to say no when asked if I can fill in.	**0**.**96**	−0.02	0.02	−0.03	0.91
	5. I feel bad for the residents if I don’t fill in.	**0**.**57**	0.00	0.07	0.16	0.46
	6. When I fill in for others and give up my free time, I feel guilty towards my family/friends.	**0**.**50**	0.14	−0.11	−0.12	0.26
F2: Self-endangering behavior	7. In the last four weeks I have been asked to fill in for other colleagues.	−0.02	**0**.**96**	−0.01	−0.02	0.90
	8. For the past four weeks, I've been filling in on my time-off days.	−0.01	**0**.**80**	−0.02	0.14	0.69
	9. In the last four weeks, I have had to switch shifts at short notice (e.g., instead of an early shift, I took on a late shift the next day).	0.10	**0**.**61**	0.12	−0.12	0.42
F3: Self-endangering expectations	10. In order to take good care of the residents, my colleagues should put their own physical and mental health second.	0.14	0.02	**0**.**73**	−0.07	0.57
	11. If a shift needs to be covered, I expect my colleagues to step in, even if they have to change private plans to do so.	−0.07	−0.03	**0**.**93**	0.11	0.91
	12. When staff is absent, my colleagues should step in to save other colleagues’ weekends/time off.	0.03	0.08	**0**.**70**	−0.07	0.49
F4: Self-endangering behavior tendencies	13. If a shift needs to be covered, I would fill in to make sure the residents are well taken care of.	−0.01	0.00	−0.05	**0**.**79**	0.58
	14. If a shift needs to be covered, I would fill in, even if it means changing private plans.	0.06	0.04	0.08	**0**.**93**	0.99
	Eigenvalue	3.95	1.99	2.00	1.64	
	Proportion of total variance	0.28	0.14	0.14	0.12	

The bold values makes it easier to identify the item's relationship to the factor.

### Confirmatory factor analysis

Due to the different results of PA and the CD approach, we performed CFA for the four and the three factors solution (Table 3). The four factors solution (RMSEA 90% interval = 0.046–0.085, *df* = 71.000, *χ*^2 ^= 123.568, CFI = .954, SRMR = .066) showed a better fit than the three factors solution (RMSEA 90% interval = 0.091–0.123, *df* = 74.000, *χ*^2 ^= 218.273, CFI = .875, SRMR = .087). A model comparative ANOVA showed that the three-factor model fit significantly worse than the four-factor model, *χ*^2 ^= 218.27, *p* < .001, Δ *AIC *= 88.7.

**Table 3:** Results of confirmatory factor analysis for the relationships among the facets of self-endangering (after deletion of three items due to crossloadings above 32).

Model	*χ* ^2^	df	CFI	TLI	RMSEA 90% interval		SRMR
	* *	* *	* *	* *	*LL*	*UL*	* *
Higher order	123.910	73.000	.956	.945	.044	.083	.067
3-factor	218.273	74.000	.875	.846	.091	.123	.087
4-factor	123.568	71.000	.952	.942	.046	.085	.066

*χ^2^*, chi-square statistic; SRMR, standardized root mean square residual; RMSEA, root mean square error of approximation; CFI, comparative fit index; TLI, Tucker-Lewis index.

After EFA and CFA, the final version of self-endangering behavior tendency consisted of two items, and self-endangering cognitions consisted of six items. Internal validity for self-endangering behavior tendency was high (2 items, *ω_total1 _*= .90). Internal validity for self-endangering cognitions was high (6 items, *ω_total1 _*= .91). See the supplemental online file for the final version of the self-endangering questionnaire.

### Analytic strategy

To test our hypotheses, we originally wanted to calculate longitudinal mediation analyses using the independent variables (e.g., altruism, team identification, and self-worth) from wave 1, the mediation variables (e.g., subscales of self-endangering) from wave 2, and the dependent variable (e.g., burnout exhaustion) from wave 3. An *a priori* power analysis resulted in a sample size of 138. Due to the global COVID-19 pandemic in 2020 and 2021, our sample size decreased from wave one with 416 participants (July to August 2020) to 35 participants in wave three (January to March 2021). Therefore, a longitudinal evaluation including all three measurement points was no longer advisable.

In order to still gain maximum information from the collected data, we used a multiverse analytic strategy (53). We selected two approaches to test our hypothesis: (1) regression analysis with measurement repetition, and (2) cross-lagged panel design for waves 1 and 2. We conducted all analyses with RStudio Version 1.3.959 (54).

We provide descriptive statistics for the variables that describe the sample and the workstrain context of the participants in more detail in an additional online appendix: workload, emotional dissonance, psychological detachment, and self-care. Further, we describe descriptive statistics for the independent variables (IV), dependent variables (DV) and mediation variables (MV). To test hypothesis, we provide regression analysis statistics and path analysis models for variables: IV—altruism, team identification, self-esteem; DV—exhaustion and disengagement; and mediators—self-endangering cognitions and behavior tendencies.

## Results

### Descriptive statistics

Table 4 shows descriptive statistics and correlations among independent and dependent variables and mediators.

**Table 4:** Descriptive statistics and correlations Among independent and dependent variables and mediators From analysis sample From Measurement time 1*.*

Variable	*M*	*SD*	1	2	3	4	5	6
1. Altruism	3.84	0.67						
2. Team identification	4.92	1.35	.22**					
			[.09,.35]					
3. Self-esteem	3.71	1.01	.04	.08				
			[−.09,.18]	[−.06,.21]				
4. Self-endangering behavior tendency	3.49	1.10	.36**	.18**	−.03			
			[.24,.47]	[.05,.31]	[−.17,.10]			
5. Self-endangering cognitions	2.94	1.03	.27**	.03	−.42**	.33**		
			[.14,.39]	[−.11,.17]	[−.52, −.30]	[.20,.44]		
6. Burnout Exhaustion	2.54	0.57	.07	−.14*	−.36**	−.04	.40**	
			[−.07,.20]	[−.27, −.01]	[−.47, −.23]	[−.17,.10]	[.28,.51]	
7. Burnout disengagement	2.08	0.57	−.04	−.18**	−.32**	−.19**	.28**	.67**
			[−.18,.09]	[−.31, −.05]	[−.43, −.19]	[−.32, −.06]	[.15,.40]	[.59,.74]

*M* and *SD* are used to represent mean and standard deviation, respectively. Values in square brackets indicate the 95% confidence interval for each correlation. The confidence interval is a plausible range of population correlations that could have caused the sample correlation (Cumming, 2014).

* Indicates *p* < .05.

** Indicates *p* < .01.

### Regression analysis

To test hypotheses 1a—6b we computed a multiple regression model with repeated measurements. Due to the longitudinal design, the model had two levels, with individuals' repeated measurements nested in individuals. We followed recommendations by Bliese (2002, 2016) (55, 56) and calculated intraclass correlations (ICCs) (57) to test for potential non-independence justified by the hierarchical structure of the data, for exhaustion and disengagement (dependent variables) and self-endangering cognitions and behavior tendency (mediating variables).

Exhaustion was non independent in individuals, ICC(1) = .56, *F*(215, 75) = 2.75, *p* < .001. Individuals were also somewhat distinguishable by their average level of exhaustion, ICC(2) = .63. Disengagement was non independent in individuals, ICC(1) = .66, *F*(215, 75) = 3.60, *p* < .001. Individuals were also distinguishable by their average level of disengagement, ICC(2) = .72. Self-endangering cognitions were non independent in individuals, ICC(1) = .67, *F*(214, 73) = 3.78, *p* < .001. Individuals were also distinguishable by their average level of self-endangering cognition, ICC(2) = .73. Self-endangering behavior tendency was non independent in individuals, ICC(1) = .71, *F*(215, 77) = 4.40, *p* < .001. Individuals were also distinguishable by their average level of self-endangering behavior tendency, ICC(2) = .77. We tested our hypotheses with multilevel modeling.

We explored the random effect structure by testing random intercept models for the dependent variables exhaustion and disengagement and the mediators self-endangering cognitions and behavior tendency and measurement time as the independent variable. The calculation of random intercept and slope models was not possible, because too few people participated in several measurement points. We therefore tested the hypotheses with random intercept models using mean centered predictor variables.

### Hypotheses tests using mixed models

Hypothesis 1 proposed that altruistic job motivation is positively related to self-endangering cognitions and self-endangering behavior tendency. To test, we investigated model 3 and 4; see Table 5. Tests revealed a significant effect of altruism on self-endangering cognitions, *b* = 0.47, 95% CI = [0.30, 0.63], and on self-endangering behavior tendency, *b* = 0.54, 95% CI = [0.36, 0.71].

**Table 5:** Mixed models regressing repeated within-person measures of dependent variables and mediators on study variables.

	Model 1: DV = Burnout exhaustion				Model 2: DV = Burnout disengagement				Model 3: DV = Self-endangering cognitions				Model 4: DV = Self-endangering behavior tendency			
	b	SE	95% CI		b	SE	95% CI		b	SE	95% CI		b	SE	95% CI	
Direct Effect			LL	UL	** **	** **	LL	UL	** **	** **	LL	UL	** **	** **	LL	UL
Fixed effects																
Intercept	2.53	0.03	2.46	2.60	2.08	0.03	2.01	2.15	2.91	0.06	2.79	3.04	3.44	0.06	3.31	3.58
Altruism	0.07	0.05	−0.01	0.17	−0.02	0.04	−0.11	0.07	**0**.**47**	**0**.**08**	**0**.**30**	**0**.**63**	**0**.**54**	**0**.**08**	**0**.**36**	**0**.**71**
Team identification	−**0**.**05**	**0**.**02**	−**0**.**10**	−**0**.**009**	−**0**.**06**	**0**.**02**	−**0**.**11**	−**0**.**02**	−0.02	0.04	−0.10	0.05	**0**.**11**	**0**.**04**	**0**.**02**	**0**.**19**
Self-esteem	−**0**.**18**	**0**.**03**	−**0**.**25**	−**0**.**11**	−**0**.**16**	**0**.**03**	−**0**.**23**	−**0**.**10**	−**0**.**39**	**0**.**05**	−**0**.**50**	−**0**.**28**	−0.01	0.06	−0.14	0.09
Time	0.05	0.41	−0.02	0.14	0.02	0.03	−0.04	0.10	0.09	0.06	−0.02	0.22	−0.03	0.06	−0.16	0.09
Random effects																
Variance components																
Level 1 (Within-person)	0.14				0.15				0.51				0.69			
Level 2 (Between-person)	0.13				0.11				0.29				0.28			
Observations	283				283				279				281			
Deviance (-2LogLik)	419.8				401.5				690.8				734.0			
AIC	433.8				415.5				704.8				748.0			
BIC	459.3				441.0				730.2				773.5			
Pseudo-R²	0.13				0.12				0.23				0.14			

Bold indicates the significant value.

Confidence Intervals are based on 10,000 bootstrapped samples. Predictor variables are mean centered.

Hypothesis 2 proposed that team identification is positively related to self-endangering cognitions and self-endangering behavior tendency. To test, we investigated model 3 and 4; see Table 5. There was no significant effect of team identification on self-endangering cognitions, *b* = −0.02, 95% CI = [−0.10, 0.05], but a significant effect of team identification on self-endangering behavior tendency, *b* = 0.11, 95% CI = [0.02, 0.19].

Hypothesis 3 proposed that self-esteem is negatively related to self-endangering cognitions and self-endangering behavior tendency. To test, we investigated model 3 and 4; see Table 5. There was a significant negative effect of self-esteem on self-endangering cognitions, *b* = −0.39, 95% CI = [−0.50, −0.28], but no significant effect of self-esteem on self-endangering behavior tendency, *b* = −0.01, 95% CI = [−0.14, 0.09].

Hypothesis 4 proposed that self-endangering cognitions and behavior tendency are positively related to emotional exhaustion. To test, we investigated model 5, see Table 6. This revealed a significant positive effect of self-endangering cognitions on emotional exhaustion, *b* = 0.19, 95% CI = [0.12, 0.26], and a significant negative effect of self-endangering behavior tendency on emotional exhaustion, *b* = −0.08, 95% CI = [−0.14, −0.01].

**Table 6:** Mixed models regressing repeated within-person measures of dependent variables on mediators and independent variables.

	Model 5: DV = Burnout exhaustion				Model 6: DV = Burnout disengagement			
	b	SE	95% CI		b	SE	95% CI	
Direct Effect			LL	UL	** **	** **	LL	UL
Fixed effects								
Intercept	2.53	0.03	2.46	2.60	2.08	0.03	2.01	2.15
Altruism	0.01	0.05	−0.08	0.12	−0.02	0.05	−0.12	0.07
Team identification	−0.05	0.02	−0.09	0.001	−**0**.**04**	**0**.**02**	−**0**.**09**	−**0**.**001**
Self-esteem	−**0**.**12**	**0**.**03**	−**0**.**19**	−**0**.**04**	−**0**.**11**	**0**.**03**	−**0**.**18**	−**0**.**04**
Time	0.05	0.41	−0.02	0.14	0.01	0.03	−0.04	0.10
Self-endangering cognitions	**0**.**19**	**0**.**03**	**0**.**12**	**0**.**26**	**0**.**15**	**0**.**03**	**0**.**08**	**0**.**22**
Self-endangering behavior tendency	−**0**.**08**	**0**.**03**	−**0**.**14**	−**0**.**01**	−**0**.**14**	**0**.**03**	−**0**.**21**	−**0**.**08**
Random effects								
Variance components								
Level 1 (Within-person)	0.14				0.14			
Level 2 (Between-person)	0.10				0.10			
Observations	283				279			
Deviance (-2LogLik)	419.8				369.1			
AIC	433.8				387.1			
BIC	459.3				419.8			
Pseudo-R²	0.13				0.12			

Bold indicates the significant value. Confidence Intervals are based on 10,000 bootstrapped samples. Predictor variables are mean centered.

Hypothesis 5a/b proposed that self-endangering cognitions/behavior tendency mediate the relationship between altruistic job values and burnout. To test, we investigated mediation modeling; see Table 7. We found a significant indirect effect from altruism over self-endangering cognitions to exhaustion, b = 0.092, 95% CI = [0.04; 0.14] and from altruism over self-endangering behavior tendency to exhaustion, b = −0.044, 95% CI = [−0.08; −0.01].

**Table 7:** Unstandardized coefficients, confidence intervals and *p* values of mixed models for mediation effect*s.*

Indirect effects				
Effect	Estimate	95% CI		*p*
		*LL*	*UL*	
Altruism → Self-endangering cognitions → Exhaustion	0.092	0.049	0.14	<.001***
Team Identification → Self-endangering cognitions → Exhaustion	−0.005	−0.022	0.01	.47
Self-esteem → Self-endangering cognitions → Exhaustion	−0.075	−0.113	−0.04	<.001***
Altruism → Self-endangering behavior tendency → Exhaustion	−0.044	−0.087	−0.01	.016*
Team Identification → Self-endangering behavior tendency → Exhaustion	−0.008	−0.020	0.00	.027*
Self-esteem → Self-endangering behavior tendency → Exhaustion	0.001	−0.009	0.01	.787
Altruism → Self-endangering cognitions → Disengagement	0.071	0.034	0.12	<.001***
Team Identification → Self-endangering cognitions → Disengagement	−0.004	−0.017	0.01	.479
Self-esteem → Self-endangering cognitions → Disengagement	−0.058	−0.092	−0.03	<.001***
Altruism → Self-endangering behavior tendency → Disengagement	−0.080	−0.125	−0.04	<.001***
Team Identification → Self-endangering behavior tendency → Disengagement	−0.015	−0.031	0.00	.009**
Self-esteem → Self-endangering behavior tendency → Disengagement	0.002	−0.014	0.02	.78

Confidence Intervals are based on 10,000 bootstrapped samples.

* Indicates *p* < .05.

** Indicates *p* < .01.

*** Indicates *p* < .001.

Hypothesis 6a/b proposed that self-endangering cognitions/behavior tendency mediate the relationship between team identification and burnout. To test, we investigated mediation modeling; see Table 7. We found no significant indirect effect from team identification over self-endangering cognitions to exhaustion, b = 0.005, 95% CI = [−0.022; 0.01], but a significant indirect effect from team identification over self-endangering behavior tendency to exhaustion, b = −0.008, 95% CI = [−0.02; 0.00].

### Hypothesis tests using path analysis models

#### Cross-sectional data

The tested model showed that the model had been exactly identified (*df *= 0), which is why we could not determine the model fit. We found a significant positive association between altruism and self-endangering cognitions (*b* = 0.43, *p *< .001) and self-endangering behavior tendency: More altruism led to more self-endangering cognitions and behavior tendency. Hypothesis 1a and 1b were thus confirmed.

We found non-significant associations between team identification and self-endangering cognitions (*b* = 0.002, *p *= .97) and self-endangering behavior tendency (*b* = 0.09, *p *= .06). Hypotheses 2a and 2b were not confirmed.

Hypothesis 3a was confirmed, as we found a negative significant association between self-esteem and self-endangering cognitions (*b* = −.43, *p *< .001): Lower self-esteem led to more self-endangering cognitions. We found a non-significant association between self-esteem and self-endangering behavior tendency (*b* = −.05, *p *= .43). Hypothesis 3b had to be rejected.

Hypothesis 4a and 4b proposed that self-endangering cognitions and behavior tendency are positively related to emotional exhaustion. Hypothesis 4a was confirmed. We found a significant positive association between self-endangering cognitions and exhaustion, (*b* = 0.20, *p *< .001), but a significant negative association between self-endangering behavior tendency and exhaustion (*b* = −.09, *p *= .01). This means that more self-endangering cognitions led to more exhaustion and that less self-endangering behavior tendency led to more exhaustion.

Hypothesis 5a and 5b proposed that self-endangering cognitions and behavior tendency mediate the relationship between altruistic job values and burnout. The analysis revealed that the hypothesized positive indirect effect of altruism on exhaustion mediated by self-endangering cognitions was significant (*b* = 0.09, *p *= .003) and contrary to our hypothesis we found a negative indirect effect of altruism on exhaustion mediated by self-endangering behavior tendency was significant (*b* = −.04, *p *= .04).

Hypothesis 6a and 6b proposed that self-endangering cognitions and behavior tendency mediate the relationship between team identification and exhaustion. The analysis revealed that the hypothesized positive indirect effect of team identification on exhaustion mediated by self-endangering cognitions was not significant (*b* = 0.00, *p *= .97). Further, the analysis revealed that the hypothesized positive indirect effect of team identification on exhaustion mediated by self-endangering behavior tendency was not significant (*b* = 0.01, *p *= .13).

**Figure 1:**
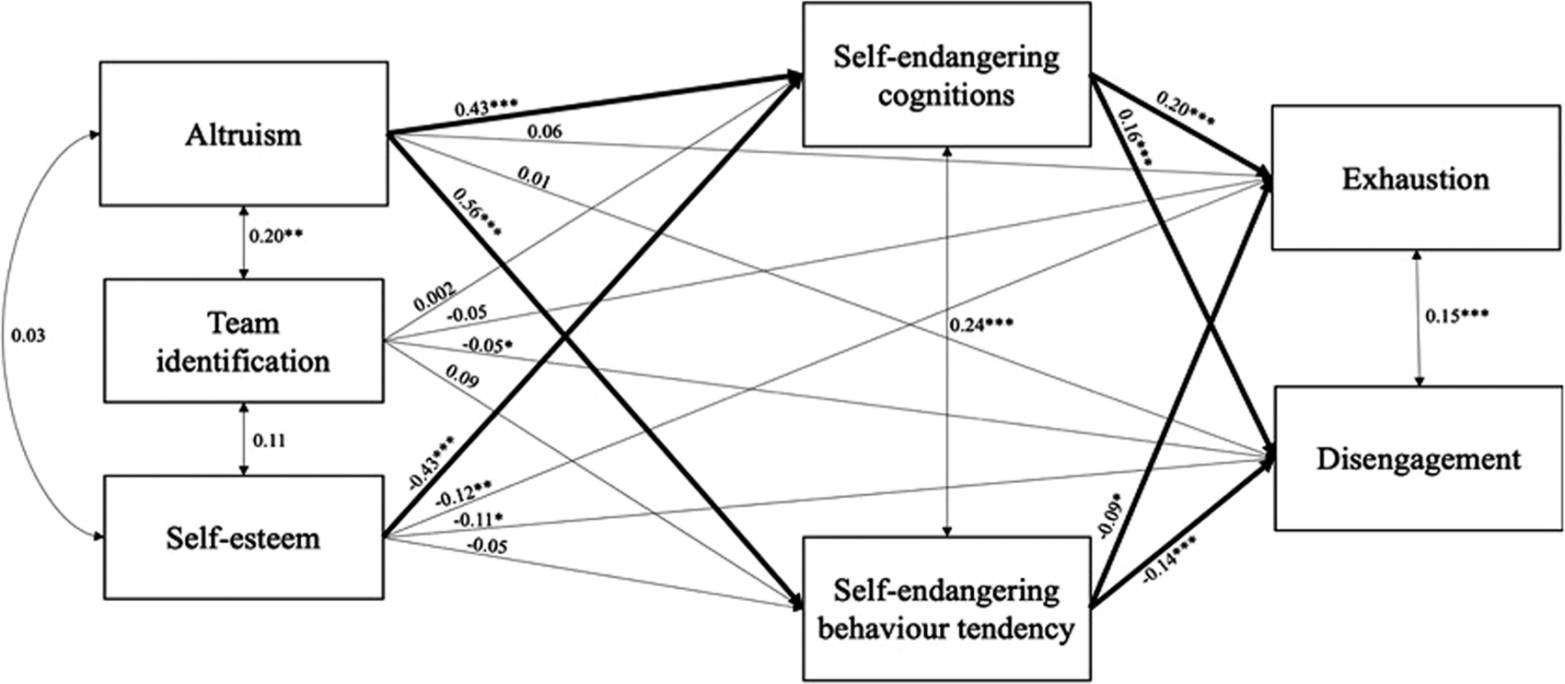
Cross sectional hypotheses testing using path analysis models (*N* = 217). Bootstrapped 10,000 times. * indicates *p* < .05. ** indicates *p* < .01. *** indicates *p* < .001.

Additionally, we found a significant indirect effect of self-esteem on exhaustion mediated by self-endangering cognitions (*b* = −.08, *p *= .001) (Figure 1).

#### Longitudinal data

**Figure 2:**
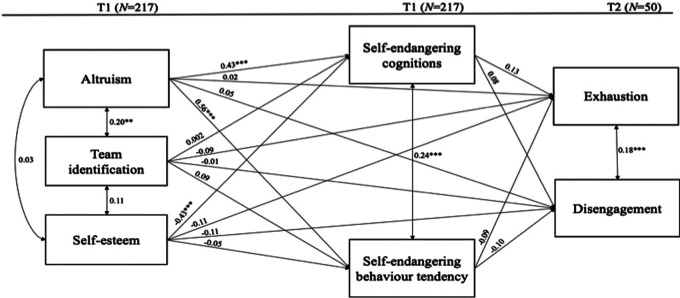
Longitudinal hypotheses testing using path analysis models. Bootstrapped 10,000 times. * indicates *p* < .05. ** indicates *p* < .01. *** indicates *p* < .001.

The tested model showed that the model had been exactly identified (*df* = 0), which is why we could not determine the model fit. The same as with the cross-sectional data, hypotheses 1a, 1b, and 3a were confirmed, and hypotheses 2a, 2b, 3b, and 4b were rejected. Again, hypotheses 4a was confirmed: Self-endangering cognitions were positively related to emotional exhaustion and behavior tendency negatively to emotional exhaustion. In contrast to the cross-sectional data, the mediation hypotheses were not confirmed in the longitudinal design (Figure 2).

## Discussion

Our study investigated the effects of altruism, team identification, and self-esteem on exhaustion mediated by self-endangering cognitions and self-endangering behavior tendency in nurses working in elderly care in Germany. Our findings show that high altruism, regardless of the analysis method and the use of longitudinal or cross-sectional data, leads, in line with hypothesis 1a and 1b, to more self-endangering cognitions and to a higher self-endangering behavior tendency. Mixed model analysis and cross-sectional path analysis also confirmed the assumed mediation effects from altruism over self-endangering cognitions to exhaustion (hypothesis 5a). In contrast to a previous research finding that high intrinsic and altruistic work values reduced nurses' burnout (11), based on our findings there is a major health risk for nurses with high altruistic values and low ability to say no under the given circumstances in care. If the care system made it possible to care for the residents in line with nurses' own “helping values”, we believe an intrinsic and altruistic motivation could improve the level of identification and meaningfulness in work. Given the terrible nursing staff situation in care, the development of a reliable plan for covering staff absences is currently unavoidable. A system has developed in which caregivers constantly experience moral imbalance and at the same time feel compelled not to leave their team and the residents in the lurch. In line with research on negative impacts of moral distress, e.g., moral distress increases burnout and dissatisfaction (58, 59), we assume there are heavy burdens on mental health, especially for this group of professionals, who show a high willingness to take on responsibility (60). We believe there is a great need to develop the skill of detached concern, defined as an emotion-regulating individual resource that leads to finding a balance between empathic concern and sufficient detachment (61). Detached concern could help nurses experience themselves as self-effective and the work as meaningful and above all increase their well-being (61). Contrary to assumptions (hypothesis 5b), longitudinal regression and cross-sectional path analysis and revealed an indirect negative effect of altruism on exhaustion mediated by self-endangering behavior tendency. We can imagine that as all analysis revealed negative relations for the subscale self-endangering behavior tendency one reason for the contradictory findings lies within the operationalization of the subscale. As we only could measure behavior tendency and not the actual behavior, e.g., frequency of actual substitution and actual overtime, we could not examine possible effects by, for example, reduced opportunities for recovery which then also increase exhaustion. Additionally, we can imagine, that, in particular, the cognitive facet of self-endangering has harmful effects, rather than the behavior or behavior tendencies, because since, for example, a bad conscience can arise even if nurses fill in, in case of absenteeism.

Considering hypothesis 2, we could only find some initial indications for positive effects from team identification to self-endangering behavior tendency: Stronger team-identification leads to a higher behavior tendency in our mixed model. Contrary to our hypothesis, self-endangering behavior tendency is negatively related to exhaustion, which means that a lower behavior tendency leads to higher exhaustion. This finding is contradictory, as we expected that a high moral commitment to the team would lead to a greater willingness to fill in for colleagues, but that this would also lead to more exhaustion. We suspect that one explanation lies in the operationalization of the construct of team-identification which only include one item. We can imagine that the wording of this item does not fit enough for the context of nursing. As analyzed in previous research (blinded for peer-review) nurses might feel high sense of duty towards their colleagues and are very sensitive to the extra work that is required for colleagues. However, nurses might not talk about “identifying” with their team or colleagues rather than feeling responsible.

Additionally, again the operationalization of the construct self-endangering behavior tendency might be difficult. Originally, we wanted to test this hypothesis using actual behavior, e.g., frequency of filling in for others, because it is obvious that people who fill in more often are working more, have less time for recovery, and are therefore more vulnerable to exhaustion. Further research should investigate this hypothesis using actual behavior frequency in a longitudinal study design. As we know that detached concern can lead to better health, nurses' identification with residents and patients should be investigated in future studies to identify possible associations with self-endangering. We could not find distinct results about the effect for the assumed mediation effect of self-endangering cognitions/behavior on the relation between team-identification and exhaustion (hypothesis 6a/b). First, considering our results it is still unclear how team-identification affects exhaustion, which is why further more complex analyses are less useful. Second, we can imagine that other team-based variables influence the effect of team identification on health, such as team commitment and psychological safety (62). Psychological safety climate promotes employees' wellbeing (63), so we assume that psychological safety also reduces self-endangering cognitions and behavior. Moreover, we can imagine that only identifying with colleagues might lead to self-endangering because identification alone might be not enough to feel secure in the team and therefore to be able to communicate one's boundaries. Future research should measure team identification and psychological safety and should investigate possible interaction effects.

Additionally, in line with our hypothesis 3, we found that low self-esteem leads to more self-endangering cognitions. Mixed model analysis and cross-sectional path analysis also showed a mediation effect from self-esteem over self-endangering cognitions to exhaustion: Low self-esteem promotes more self-endangering cognitions, and this leads to stronger feelings of exhaustion. Previous research found that nurses with high self-esteem have fewer psychological problems (64), cope more effectively in stressful situations (33, 65), and are more active and flexible (33). Taking these findings into account and bringing them together with our results, we consider that the mediation over self-endangering cognitions, e.g., nurses not feeling able to say no when asked to fill in for another nurse and having a guilty conscience when they do not fill in, could explain the psychological mechanism between self-esteem and exhaustion. We can also imagine that nurses with low self-esteem may identify themselves strongly with their work, e.g., their relationship with residents, the feeling of being needed, and the feelings they get from helping and guiding them, such that these experiences confirm them in their identity and self. This would result in nurses being “quasi-dependent” on their work for their self-esteem, making it difficult for them to say no, and when they do say no, feeling guilty about it.

Independent from the analysis method we found, that more self-endangering cognitions lead to more exhaustion (hypothesis 4a) and contrasting to our assumption (hypothesis 4b) that more self-endangering behavior tendency leads to less exhaustion. These results suggest that in particular self-endangering cognitions tend to have harmful effects and are exhausting over time. Recent research should investigate if those employees that tend to show self-endangering behavior tendency are also those employees that do fill in in case of absenteeism or if it is just a tendency that does not result in actual behavior which would mean that they do not have much overtime and therefore time for recovery and less risk for exhaustion.

## Theoretical implications

In view of the contradictory findings in the research on the effect of altruism on the experience of exhaustion it seems necessary to examine whether there is an optimal level of altruistic motivation, e.g., an inverse u-shaped relation. We can also imagine that altruistic motivation, similarly to self-regulatory abilities, can be exhausted over time (66) and that nurses constantly experience a goal discrepancy with their altruistic motives, which promotes exhaustion.

Taking into account that self-esteem should be differentiated into self-liking and self-competence (67), especially the facet of self-liking might be interesting to examine in the context of self-endangering. Future research should investigate which facet explains more variance in self-endangering.

Servant leadership is related to meaning and work engagement (68) and organizational citizenship behavior and performance (69), so we assume that leadership behavior could have a similar influence on self-endangering, in that leaders who tend to ask the same employees for filling in may promote self-endangering in nursing.

As the data in subset A (used for exploratory and confirmatory data analysis) did not meet the criteria for normally distributed data, we consider that further research is needed to validate the self-endangering' items. We assume that due to the highly demanding working conditions nurses will always tend to show high rates of self-endangering, which is why normally distributed data is probably unlikely to find in this specific occupational group. Nevertheless, further research is needed to increase the significance of our self-endangering' items for the context of nursing.

## Practical implications

Based on our results we believe that person-focused interventions could help to increase nurses' self-esteem and help them to develop a healthy and balanced altruistic job motivation. Additionally, leaders should be sensitized to notice if an employee tends to show too much self-endangering.

Further, fundamental changes in nurses’ working conditions are needed, such that a reliable work schedule, using validated patient-to-nurse ratios, and a standardized absenteeism plan build the basis of the nursing work. From our point of view, this can be achieved by investing more money in nursing staff, so that that absences can be covered by external staff and the profession is made more attractive to young people. For example, more money could be provided to the care system, if a “care fund”, similar to the old “solidarity tax” in Germany, were set up to cope with demographic change.

## Limitations

To our knowledge this is the first quantitative study that focuses on individuals' self-endangering cognitions and behavior among nurses in the field of long-term care. Of course, our study is not without limitations, especially because our longitudinal evaluation was severely limited by the small sample size. Our sample decreased over the two waves from 416 to 50. We can image that in particular the worldwide COVID-19 pandemic causes this participant loss, as nurses suffered under high work strain (2, 4) and therefore the motivation for extra work, like participating in a survey without having any advantages, might decrease. In times of highly exhausted nurses (3) the willingness for extra tasks is certainly limited. Additionally, we assume that by participating in this survey, individuals might be confronted with their situation in the health care system, which might in particular worrying in times of a pandemic, where feelings of insecurity might be high. It should also be considered that the second measurement point, was around the time of the beginning of COVID-19 vaccinations which we can imagine lead to nurses' insecurity and anxiety and perhaps also to a reluctance to engage in scientific research.

First, our results are not generalizable and need to be examined in a large longitudinal design. It is also critical to note that we developed the items for self-endangerment ourselves, so future research should validate the items of self-endangering in a large sample.

Second, our sample is not representative and does not include data on nurses in acute care settings, e.g., hospitals, and due to the small sample size, we could not differentiate between outpatient and stationary care. In the future, group comparisons between different care settings should be examined. We can also imagine that having a child could influence the development of self-endangering; future research should investigate this aspect.

Third, as the data for EFA and CFA did not meet the criteria for normally distributed data the results cannot be generalized for other occupational groups and, therefore, further studies on larger samples and, in comparison, also on less strained samples are needed, especially for conclusions on other occupational groups. In large validation studies, the items should be tested for scale validity.

Nevertheless, we believe that our study offers new knowledge and approaches in the context of the health of workers in elderly care.

## Conclusions

This quantitative study dealt with the question of self-endangering in nursing and possible antecedents and mediator effects. Our results show that a high altruistic job motivation and low self-esteem can lead to self-endangering and that this in turn can promote exhaustion in nurses working in elderly care. Future research needs to investigate whether data on nurses in hospitals confirm these assumptions. Our results underline the great need to change working conditions in nursing in a way that promotes stability in staffing.

## Data Availability

The raw data supporting the conclusions of this article will be made available by the authors, without undue reservation.

## Supplementary material

Supplementary Data Sheet S1 Appendix 1: questionnaire

Supplementary Data Sheet S2 Appendix 2: context Information
