# Supplementary material for: The role of self-endangering cognitions between long-term care nurses' altruistic job motives and exhaustion
Source: Front Health Serv. 2023 Aug 23;3:1100225. doi: 10.3389/frhs.2023.1100225 (PMC10482104; doi:10.3389/frhs.2023.1100225)
Supplement: Supplementary Data Sheet S1 — Appendix 1: questionnaire [file Datasheet1.docx]

**Appendix 1: questionnaire**

Final version of the self-endangering questionnaire (scales used for the study: self-endangering behavior tendency and cognitions)

| Subfacets | Item |  |  |  | |  | |  | | | | |
| --- | --- | --- | --- | --- | --- | --- | --- | --- | --- | --- | --- | --- |
|  |  | Not true at all | | | | |  | | | Absolutely true | | |
| Self-endangering behavior tendency | 1. If a shift needs to be covered, I would fill in to make sure the residents are well taken care of. | 1 | | | 2 | | | | 3 | | 4 | 5 |
|  | 2. If a shift needs to be covered, I would fill in, even if it means changing private plans. | 1 | | | 2 | | | | 3 | | 4 | 5 |
| Self-endangering behavior | 3. In the last four weeks I have been asked to fill in for other colleagues. | 1 | | | 2 | | | | 3 | | 4 | 5 |
|  | 4. For the past four weeks, I've been filling in on my time-off days. | 1 | | | 2 | | | | 3 | | 4 | 5 |
|  | 5. In the last four weeks, I have had to switch shifts at short notice (e.g., instead of an early shift, I took on a late shift the next day). | 1 | | | 2 | | | | 3 | | 4 | 5 |
| Self-endangering cognitions | 6. I find it difficult to say no to my leaders when I have to fill in for other colleagues. | 1 | | | 2 | | | | 3 | | 4 | 5 |
|  | 7. I find it difficult to say no to my colleagues when I have to fill in for them. | 1 | | | 2 | | | | 3 | | 4 | 5 |
|  | 8. I have a hard time saying no on the phone when asked if I can fill in. | 1 | | | 2 | | | | 3 | | 4 | 5 |
|  | 9. I find it difficult in personal contact (e.g., directly at work) to say no when asked if I can fill in. | 1 | | | 2 | | | | 3 | | 4 | 5 |
|  | 10. I feel bad for the residents if I don’t fill in. | 1 | | | 2 | | | | 3 | | 4 | 5 |
|  | 11. When I fill in for others and give up my free time, I feel guilty towards my family/friends. | 1 | | | 2 | | | | 3 | | 4 | 5 |
|  | 12. In order to take good care of the residents, my colleagues should put their own physical and mental health second. | 1 | | | 2 | | | | 3 | | 4 | 5 |
| Self-endangering expectations | 13. If a shift needs to be covered, I expect my colleagues to step in, even if they have to change private plans to do so. | 1 | | | 2 | | | | 3 | | 4 | 5 |
|  | 14. When staff is absent, my colleagues should step in to save other colleagues’ weekends/time off. | 1 | | | 2 | | | | 3 | | 4 | 5 |
