# Supplementary material for: The role of self-endangering cognitions between long-term care nurses' altruistic job motives and exhaustion
Source: Front Health Serv. 2023 Aug 23;3:1100225. doi: 10.3389/frhs.2023.1100225 (PMC10482104; doi:10.3389/frhs.2023.1100225)
Supplement: Supplementary Data Sheet S2 — Appendix 2: context Information [file Datasheet2.docx]

**Appendix 2: Context Information**

*Descriptive statistics*

We surveyed various additional data, e.g., variables describing workload and psychological work strain: Workload, emotional dissonance, psychological detachment and self-care.

The qualitative as well as quantitative workload was relatively stable across the three measurement points for acute care and stationary care settings. For both settings, the quantitative workload was higher than the qualitative workload. Also, emotional dissonance, psychological detachment, and self-care did not change, except for minor changes, over time (Appendix Table 1).

**Appendix Table 1**

*Means and standard deviations among study variables for measurement times 1,2 and 3*

|  |  | T1 | |  | T2 | |  | T3 | |
| --- | --- | --- | --- | --- | --- | --- | --- | --- | --- |
| Variable |  | *M* | *SD* |  | *M* | *SD* |  | *M* | *SD* |
|  |  |  |  |  |  |  |  |  |  |
| Qualitative workload stationary care ^1^ |  | 2.55 | 0.87 |  | 2.68 | 0.76 |  | 2.67 | 0.64 |
| Quantitative workload stationary care ^1^ |  | 3.60 | 0.80 |  | 3.68 | 0.69 |  | 3.68 | 0.76 |
| Qualitative workload ambulant care ^1^ |  | 2.20 | 0.78 |  | 2.64 | 0.99 |  | 2.33 | 0.84 |
| Quantitative workload ambulant care ^1^ |  | 3.02 | 1.03 |  | 3.38 | 0.95 |  | 3.30 | 1.15 |
| Emotional dissonance ^2^ |  | 2.66 | 1.11 |  | 2.85 | 1.06 |  | 2.83 | 1.32 |
| Psychological detachment ^3^ |  | 2.80 | 1.04 |  | 2.85 | 0.86 |  | 2.78 | 0.99 |
| Self-care ^4^ |  | 2.78 | 0.64 |  | 2.75 | 0.56 |  | 2.77 | 0.71 |
|  |  |  |  |  |  |  |  |  |  |
|  |  |  |  |  |  |  |  |  |  |

*Note.* *M* and *SD* are used to represent mean and standard deviation, respectively.

^1^ Questionnaire of Psychological Stress in hospitals/outpatient care/long-term care ("miab"; Nickel & Kersten, 2014)

^2^  Frankfurter Emotion Work Scale (Zapf et al., 1999)

^3^ FABA-Questionnaire (Richter et al., 1996)

^4^ Based on results from earlier qualitative research (Eder & Meyer, 2022) we developed 7 items around self-care in nursing.
